# Correlation of Decreased Serum Pituitary Adenylate Cyclase-Activating Polypeptide and Vasoactive Intestinal Peptide Levels With Non-motor Symptoms in Patients With Parkinson’s Disease

**DOI:** 10.3389/fnagi.2021.689939

**Published:** 2021-09-08

**Authors:** Shiyu Hu, Shen Huang, Jianjun Ma, Dongsheng Li, Zhenxiang Zhao, Jinhua Zheng, Mingjian Li, Zhidong Wang, Wenhua Sun, Xiaoxue Shi

**Affiliations:** ^1^Department of Neurology, Henan Provincial People’s Hospital, Zhengzhou, China; ^2^Department of Neurology, People’s Hospital of Henan University, Zhengzhou, China; ^3^Department of Neurology, People’s Hospital of Zhengzhou University, Zhengzhou, China

**Keywords:** Parkinson’s disease, pituitary adenylate cyclase-activating polypeptide, vasoactive Intestinal peptide, non-motor symptoms, cross-sectional study

## Abstract

**Objective:** Pituitary adenylate-cyclase activating polypeptide (PACAP) and vasoactive intestinal peptide (VIP) are two neuropeptides that exhibit anti-inflammatory and neuroprotective properties, modulating the production of cytokines and chemokines, and the behavior of immune cells. However, the relationship between PACAP and VIP levels and Parkinson’s disease (PD) are not clear. The aim of the current study was to evaluate serum PACAP and VIP levels in PD patients and to analysis the correlation between neuropeptide levels and non-motor symptoms.

**Methods:** In this cross-sectional study, we enrolled 72 patients with idiopathic PD and 71 healthy volunteers. Serum PACAP and VIP levels were measured using an enzyme-linked immunosorbent assay (ELISA) kit. Non-motor symptoms were assessed with the Non-Motor Symptoms Scale (NMSS) for PD, including total and single-item scores.

**Results:** The serum PACAP levels of PD patients were significantly lower than those of healthy controls [(76.02 ± 43.78) pg/ml vs. (154.96 ± 76.54) pg/ml, *P* < 0.001]; and the serum VIP levels of PD patients were also significantly lower than those of healthy controls [(109.56 ± 15.39) pg/ml vs. (136.46 ± 24.16) pg/ml, *P* < 0.001]. PACAP levels were inversely correlated only with the score on NMSS item five, assessing Attention/memory (*r* = −0.276, *P* < 0.05) and lower serum PACAP levels were detected in the cognitive dysfunction subgroup than in the cognitively intact subgroup [(61.87 ± 32.66) pg/ml vs. (84.51 ± 47.59) pg/ml, *P* < 0.05]; meanwhile, VIP levels were inversely correlated with the NMSS total score (*r* = −0.285, *P* < 0.05) and the single-item scores for item one, assessing Cardiovascular (*r* = −0.257, *P* < 0.05) and item three, assessing Mood/cognition (*r* = −0.373, *P* < 0.05), and lower serum VIP levels were detected in the anxiety subgroup and depression subgroup than in the non-anxiety subgroup and non-depression subgroup, respectively [(107.45 ± 15.40) pg/ml vs. (116.41 ± 13.67) pg/ml, *P* < 0.05]; [(104.45 ± 15.26) pg/ml vs. (113.43 ± 14.52) pg/ml, *P* < 0.05].

**Conclusion:** The serum PACAP and VIP levels of PD patients were significantly lower than those of healthy controls. The non-motor symptoms significantly negatively correlated with serum PACAP level was cognitive dysfunction, while mood disorder was significantly correlated with serum VIP level.

## Introduction

Parkinson’s disease (PD) is a chronic neurodegenerative disorder involving the loss of dopaminergic neurons and the formation of Lewy pathology [mainly a-synuclein (a-syn)] in the substantia nigra (Qian et al., 2020). Its typical characteristics include motor symptoms such as bradykinesia, tremor at rest, and rigidity, which commonly present along with gait impairment. At the same time, non-motor symptoms such as hyposmia, constipation, pain, cognitive dysfunction, sleep, and mood disturbances often occur as well (Garcia-Ruiz et al., 2014; Lotankar et al., 2017). Although the pathogenesis of PD is not clear, neuroinflammation, in particular glia activation, has been identified as key factors in the progression of the disease (Gelders et al., 2018; Hall et al., 2018). In neuropathological studies, activated microglia has been found close to dopaminergic neurons in PD patients and cytokines have been found in higher levels in the striatum and substantia nigra of PD brains (Garcia-Esparcia et al., 2014). Anti-inflammatory and neuroprotective effects of neuropeptides, such as pituitary adenylate cyclase-activating polypeptide (PACAP) and vasoactive intestinal peptide (VIP), have been widely demonstrated in PD models *in vivo* and *in vitro* (Joers et al., 2017; Campos-Acuna et al., 2019; Panicker et al., 2019). PACAP and VIP are two neuropeptides that are structurally related, which have three known receptors, VPAC1, VPAC2, and PAC1, that belong to the G-protein-coupled receptor (GPCR) family. The VPAC1/2 receptors recognize VIP with high affinity, while PAC1 receptor binds to PACAP with higher affinity than VIP (Moody et al., 2011). Data from experimental studies have revealed that VIP and PACAP receptors are widely distributed in the nervous, endocrine, and immune systems among others (Moody et al., 2011; Hirabayashi et al., 2018). They exhibit anti-inflammatory and neuroprotective properties, modulating the production of cytokines and chemokines, and the behavior of immune cells (de Souza et al., 2020). Furthermore, studies in cellular models revealed that PACAP and VIP Protect against Aβ-mediated toxicity in PC12 cells (Onoue et al., 2002). In animal models of PD, VIP Prevents oxidative stress and apoptosis in 6-hydroxydopamine (6-OHDA)-lesioned rats (Tuncel et al., 2012) and PACAP improves learning and memory in three different paradigms of the water-maze task in MPTP-injected mice (Onoue et al., 2002). More importantly, PACAP and VIP have been detected in plasma (Baranowska-Bik et al., 2013; Han et al., 2015; Ma et al., 2015; Jiang et al., 2016), serum (Cernuda-Morollon et al., 2016), and cerebrospinal fluid (CSF) (Gjerris et al., 1984; Baranowska-Bik et al., 2013; Han et al., 2014) of some diseases in humans, such as multiple sclerosis, Alzheimer disease, cerebral hemorrhage, migraineur, atypical depression, and et al. However, there is a dearth of data on the levels of these neuropeptides in PD patients, and there are no previous studies exploring the potential relationship between serum PACAP and VIP levels and non-motor symptoms of PD. Therefore, the purposes of this study were (1) to evaluate the levels of PACAP and VIP in the serum in PD patients and healthy controls, and (2) to investigate whether there is any correlation between neuropeptide levels and non-motor symptoms in patients with PD.

## Materials and Methods

### Participants

Patients with PD from December 2018 to October 2020 at the clinic of the Henan Provincial People’s Hospital, Henan Province, China. PD patients were all clinically diagnosed according to the International Parkinson and Movement Disorder Society (MDS) Clinical Diagnostic Criteria for the diagnosis of PD (Postuma et al., 2015). The exclusion criteria were as follows: (1) patients diagnosed with neurological disease except PD (Alzheimer disease, epilepsy, cerebrovascular disease, severe head trauma, etc.); (2) patients who might have been pregnant or breastfeeding; (3) patients who might have a history of headache; and (4) patients who had severe complications. Healthy, age- and gender-matched volunteer controls were recruited at the same time from the health examination center. A total of 71 healthy volunteers participated in this study. The exclusion criteria were applied to both patients and controls. All subjects were made aware of the contents of the study, and a written informed consent document was obtained. The research protocol was approved by the Ethics Committee of Henan Provincial People’s Hospital of Medical Science.

### Clinical Assessment

For all included participants, demographic data and medical history were recorded. Motor symptom severity was evaluated in terms of Unified Parkinson’s Disease Rating Scale (UPDRS) part III motor scores and Hoehn–Yahr (H-Y) stages. Non-motor symptoms were assessed with the Non-Motor Symptoms Scale (NMSS) (Chaudhuri et al., 2007) for PD, including total and single-item scores. Cognition was examined with the Mini-Mental State Examination (MMSE), adjusted for age and educational level. Mood symptoms were measured with the 14-item Hamilton Anxiety Rating Scale (HAMA-14) and the 17-item Hamilton Depression Rating Scale (HAMD-17). We have partitioned our data into two categories: no anxiety symptomatology (0 ≤ HAMA-14 ≤ 6) and had anxiety symptomatology (HAMA-14 ≥ 7). A higher HAMA-14 score indicates a higher level of anxiety. The optimal threshold to utilize for maximum discrimination between depressed and non-depressed PD patients was reached at a cut-off score of 13/14 for the HAMD-17 (Huang et al., 2020). Therefore, we have divided our data into two categories:little to no depression symptomatology (0 ≤ HAMD-17 ≤ 13) and mild to marked depression symptomatology (HAMD-17 ≥ 14). A higher HAMD-17 score indicates a higher level of depression. The personal levodopa equivalent daily dose (LEDD) was finally calculated for each PD patient. All scale assessments were completed once in the patient’s off period.

### Blood Sample Collection

We collected peripheral blood (5 mL) from each participant into tubes without anticoagulant between 8:00 and 9:00 AM prior to clinical assessment (12 h of fasting). Samples were allowed to clot at room temperature for 30 min before centrifugation for 15 min at 1000 × *g*. Serum samples were then divided into several aliquots and immediately stored at −80°C until assay.

### Measurement of Serum PACAP and VIP

Serum PACAP levels were measured using an enzyme-linked immunosorbent assay (ELISA) kit (HUFI01234; ELISA Genie, Dublin, Ireland). The detection range of this kit is 7.8–500 pg/ml, with a sensitivity of 4.688 pg/ml, and the intra- and inter-assay variability were <8 and <10%, respectively. Serum VIP levels were measured using an ELISA kit (EK-064-16; Phoenix Pharmaceuticals, Burlingame, CA, United States). The detection range of this kit is 0–25 ng/ml, with a sensitivity of 0.08 pg/ml, and the intra- and interassay variability were <10 and <15%, respectively. Samples were analyzed in duplicate on the same plate in accordance with the manufactures’ instructions. PACAP and VIP levels were in all cases above the limit of detection.

### Statistical Analysis

The Kolmogorov–Smirnov test and the Levene test were used to check the data for normality and homogeneity of variances. Numerical variables are expressed as the mean ± SD. Data that did not have a normal distribution are expressed as medians (interquartile ranges). Nominal variables are expressed as percentages. Two groups of normally distributed data were compared by an independent-sample Student’s *t*-test. Multiple groups of data consistent a with normal distribution and homogeneity of variance were compared by one-way analysis of variance, and a *post hoc* least significant difference (LSD) test was used to further compare differences. For variables that violated the assumptions of normality or homoscedasticity, the groups were compared with the non-parametric Mann–Whitney U test (for 2 groups) or Kruskal–Wallis test (for >2 groups). Pearson’s (or Spearman’s) correlation analysis was conducted according to whether the variables were normally distributed. Receiver operating curve (ROC) analysis and cut-off points calculation were performed to estimate diagnostic sensitivity (Se) and specificity (Sp) values of biomarkers. Statistical analysis was performed using SPSS version 22.0 (IBM Corporation, Armonk, NY, United States) and GraphPad Prism 7 (GraphPad Software, Inc., San Diego, CA, United States). *P*-value <0.05 was considered statistically significant.

## Results

### Clinical Characteristics

In this study, 72 PD patients (mean age 64.75 ± 11.75 years, 52.8% male, and 47.2% female) and 71 controls (mean age 62.08 ± 8.69 years, 52.1% male, and 47.9% female) were enrolled from the Henan Provincial People’s Hospital between December 2018 and October 2020. In the PD group, the median number of years of education was 8, and the median duration of disease was 5 years. According to the levodopa equivalent conversion formula, the median LEDD was 337.5 mg, and the average score of the third part of the UPDRS was 45.88 scores (Table 1). There were no significant differences in gender or age between PD patients and healthy controls (*P* > 0.05).

**TABLE 1:** Clinical characteristics and serum PACAP and VIP levels of participants in individual groups in the current study.

**Characteristics**	**PD (*n* = 72)**	**Controls (*n* = 71)**	***t/χ ^2^***	***P-*Value**
Age, y	64.75 ± 11.75	62.08 ± 8.69	1.540	0.126
Male, %	38 (52.8%)	37 (52.1%)	0.006	0.937
PACAP levels, pg/ml	76.02 ± 43.78	154.96 ± 76.54	7.583	<0.001
VIP levels, pg/ml	109.56 ± 15.39	136.46 ± 24.16	7.950	<0.001
Disease duration, y	5 (2,7)	NA	NA	NA
Education, y	8 (5,10.25)	NA	NA	NA
UPDRS III score	45.88 ± 18.96	NA	NA	NA
H-Y stage	2 (2,3)	NA	NA	NA
LEDD, mg/day	337.5 (200,500)	NA	NA	NA
MMSE score	25 (20.75,27)	NA	NA	NA

*Mean ± standard deviation or median (interquartile range). PACAP, Pituitary Adenylate Cyclase-activating Polypeptide; VIP, Vasoactive Intestinal Peptide; UPDRS, Unified Parkinson’s Disease Rating Scale; H-Y stage, Hoehn–Yahr stage; LEDD, levodopa equivalent daily dose; MMSE, Mini-Mental State Examination; NA, not applicable.*

### Serum PACAP and VIP Levels in PD Patients and in Healthy Controls

**FIGURE 1:**
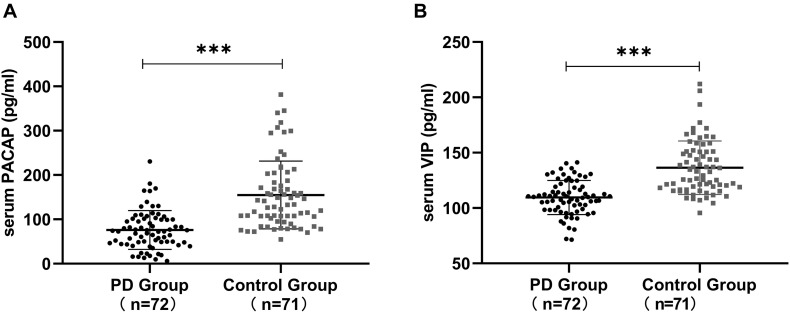
Serum PACAP and VIP levels in Parkinson’s disease (PD) patients and in healthy controls. **(A)** Lower serum PACAP levels were detected in PD patients than in healthy controls. **(B)** Lower serum VIP levels were detected in PD patients than in healthy controls. Values are presented as the mean ± standard deviation. ^∗∗∗^*P* < 0.001. PACAP, pituitary adenylate cyclase-activating polypeptide; VIP, vasoactive intestinal peptide.

Serum PACAP levels were significantly lower in PD patients than in healthy controls [(76.02 ± 43.78) pg/ml vs. (154.96 ± 76.54) pg/ml, *P* < 0.001] and serum VIP levels were significantly lower in PD patients than in the healthy controls [(109.56 ± 15.39) pg/ml vs. (136.46 ± 24.16) pg/ml, *P* < 0.001]. The differences were significant (Figure 1).

### Correlation Between Serum PACAP and VIP Levels

**FIGURE 2:**
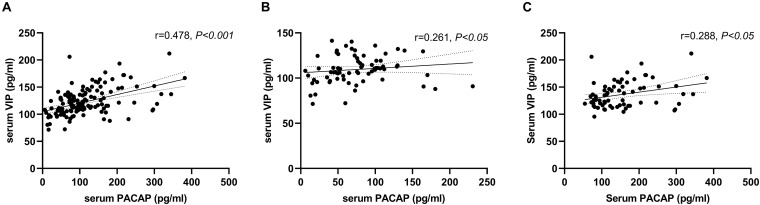
Correlation between serum pituitary adenylate-cyclase activating polypeptide (PACAP) and vasoactive intestinal peptide (VIP) levels. **(A)** A positive correlation between serum levels of PACAP and VIP in the total groups (Pearson’s correlation coefficient *r* = 0.478, *P* < 0.001, and *n* = 143). **(B)** A positive correlation between serum levels of PACAP and VIP in the PD group (Pearson’s correlation coefficient *r* = 0.261, *P* < 0.05, and *n* = 72). **(C)** A positive correlation between serum levels of PACAP and VIP in the healthy control group (Pearson’s correlation coefficient *r* = 0.288, *P* < 0.05, and *n* = 71).

We found a positive correlation between serum levels of PACAP and VIP (*r* = 0.478, *P* < 0.001). When analyzed, respectively, there was a positive correlation between serum levels of PACAP and VIP in the healthy controls (*r* = 0.288, *P* < 0.05), and there was a positive correlation in the PD Patients (*r* = 0.261, *P* < 0.05) (Figure 2).

### PACAP and VIP Levels in PD Patients at Different H-Y Stages

**FIGURE 3:**
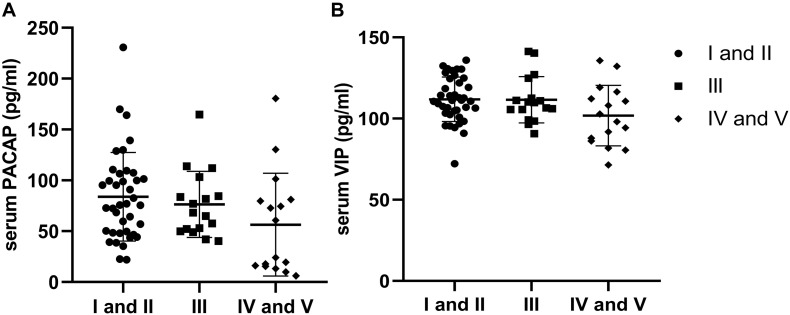
Distribution of serum levels of pituitary adenylate-cyclase activating polypeptide (PACAP) and vasoactive intestinal peptide (VIP) by Hoehn–Yahr (H-Y) stage. **(A)** There were no significant differences in the distribution of serum PACAP levels by H-Y stage in Parkinson’s disease (PD) patients (*P* > 0.05). **(B)** There were no significant differences in the distribution of serum VIP levels by H-Y stage in PD patients (*P* > 0.05).

According to the H-Y stage, PD patients were divided into stages I and II as early-stage (*n* = 39), stage III as medium-stage (*n* = 17), and stages IV and V as advanced-stage (*n* = 16). Compared to PD patients with early-stage disease, PACAP and VIP levels in the medium-stage and advanced-stage PD patients were lower, while there were no significant differences in PACAP (χ*2* = 4.828; df = 2; and *P* > 0.05) and VIP (χ^2^ = 4.158; df = 2; and *P* > 0.05) levels across the three groups (Figure 3).

### Correlation of Serum PACAP and VIP Levels With Disease Duration and Disease Severity in PD Patients

**FIGURE 4:**
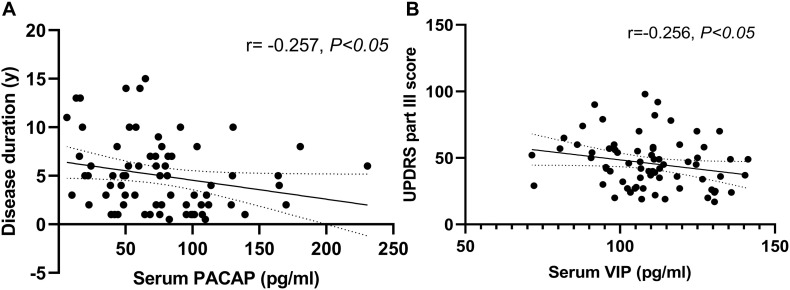
Correlation of serum pituitary adenylate-cyclase activating polypeptide (PACAP) and vasoactive intestinal peptide (VIP) levels with disease duration and disease severity in PD patients. **(A)** Serum PACAP levels were negatively correlated with disease duration (Spearman’s correlation coefficient *r* = –0.257, *P* < 0.05, and *n* = 72). **(B)** Serum VIP levels were negatively correlated with UPDRS part III scores (Pearson’s correlation coefficient *r* = –0.256, *P* < 0.05, and *n* = 72).

We found a negative correlation between serum PACAP levels and disease duration (*r* = −0.257, *P* < 0.05), and a negative correlation between serum VIP levels and UPDRS part III score (*r* = −0.256, *P* < 0.05) (Figure 4). In addition, there was no significant correlation between serum PACAP or VIP levels and LEDD.

### Receiver Operating Curve Analysis of PACAP and VIP Diagnosis of PD

**FIGURE 5:**
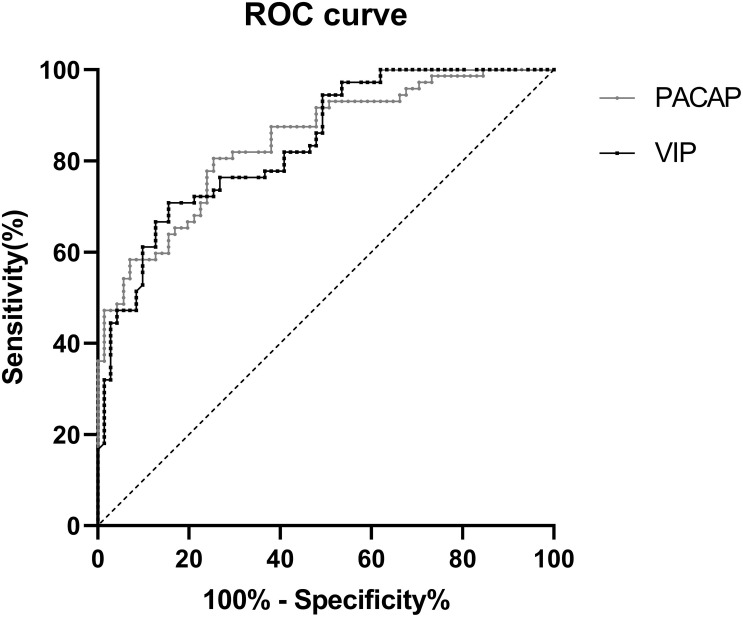
ROC curve for serum PACAP and VIP levels comparing PD group and healthy control group. ROC curve, receiver operating characteristic curve; PD, Parkinson’ s disease; AUC, area under the curve; CI, confidence interval.

Diagnostic accuracy was determined in PD patients and healthy controls by the ROC curve analysis. PACAP (AUC = 0.843; *P* < 0.001, 95% CI 0.781-0.906), at the cut-off value of 106.54 pg/ml showed 74.6% Se and 80.6% Sp. VIP (AUC = 0.838; *p* < 0.001, 95% CI 0.775-0.901), at the cut-off value of 114.90 pg/ml showed 84.5% Se and 70.8% Sp (Figure 5).

**Table:** 

	**AUC**	**95% CI**	***P-*Value**	**Cut-off value (pg/ml)**	**Sensitivity**	**Specificity**
PACAP	0.843	0.781–0.906	<0.001	106.54	74.6%	80.6%
VIP	0.838	0.775–0.901	<0.001	114.90	84.5%	70.8%

### Correlation Analysis of Non-motor Symptoms and Serum PACAP and VIP Levels in PD Patients

Correlation analysis revealed that PACAP levels were inversely correlated only with the score for NMSS item five, assessing Attention/memory (*r* = −0.276, *P* < 0.05). Additionally, VIP levels were inversely correlated with the NMSS total score (*r* = −0.285, *P* < 0.05) as well as the single-item scores for items one, assessing Cardiovascular (*r* = −0.257, *P* < 0.05) and three, assessing Mood/cognition (*r* = −0.373, *P* < 0.05). The remaining non-motor symptoms were not associated with serum PACAP or VIP levels (*P* ≥ 0.05) (Table 2).

**TABLE 2:** Correlation analysis of non-motor symptoms and serum PACAP and VIP levels in PD patients.

	**Medians (quartile ranges)**	**PACAP**		**VIP**	
		**Spearman rank**	***P***	**Spearman rank**	***P***
NMSS total score	39.50 (24.25,63.25)	–0.086	0.474	−0.285*	0.015
NMSS component score					
Cardiovascular	1.00 (0,4)	–0.163	0.170	−0.257*	0.029
Sleep/fatigue	6.00 (3,10)	–0.012	0.921	–0.159	0.181
Mood/cognition	7.00 (3,20)	0.028	0.816	−0.373**	0.001
Perceptual Problems/hallucinations	0.00 (0,1)	0.076	0.524	–0.062	0.606
Attention/memory	2.00 (2,8)	−0.276*	0.019	–0.137	0.252
Gastrointestinal tract	6.00 (2,8.75)	–0.035	0.769	–0.095	0.427
Urinary	5.50 (2,12)	–0.054	0.654	–0.003	0.980
Sexual function	0.00 (0,0)	0.011	0.930	0.107	0.373
Miscellaneous	4.00 (1,6.75)	–0.113	0.343	–0.074	0.536

NMSS, The Non-motor Symptoms Scale for Parkinson’s disease.

*P < 0.05, **P < 0.01.

Next, we used the significantly correlated non-motor symptoms as a grouping criterion to evaluate differences in PACAP and VIP levels between the two subgroups. This study included 72 patients with PD, including 27 with cognitive dysfunction (37.5%) (12 with 1–6 years of education, MMSE score <20, 10 with 7–9 years of education, MMSE score <24, and 5 with ≥10 years of education, MMSE score <26). Moreover, 55 with HAMA-14 score ≥7 who had anxiety (76.4%) and 30 with HAMA-17 score ≥14 who had depression (43.1%), 44 with NMSS item one score > 0 who had cardiovascular symptom (61.1%).

**FIGURE 6:**
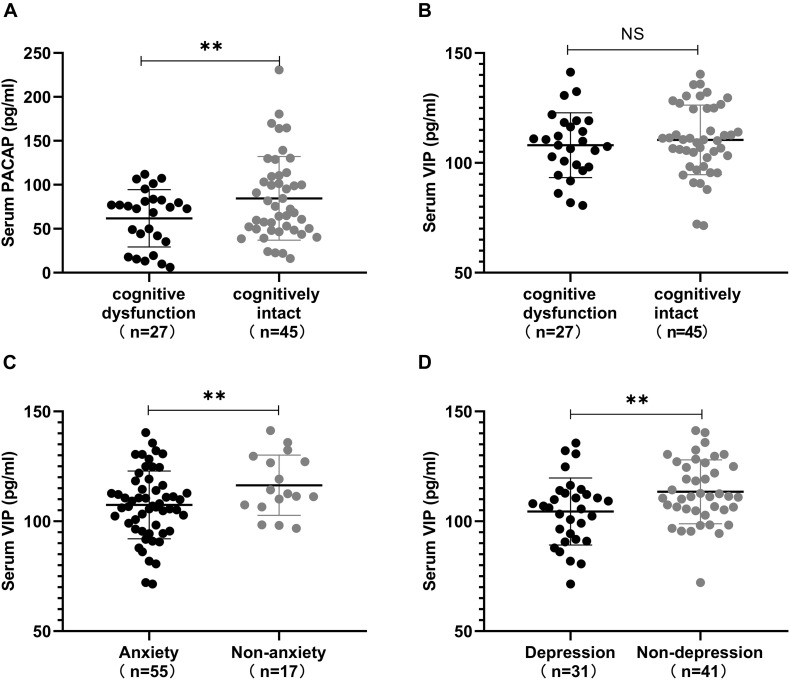
Correlation between serum pituitary adenylate-cyclase activating polypeptide (PACAP) and vasoactive intestinal peptide (VIP) levels, and non-motor symptoms. **(A)** Serum PACAP levels in different subgroups defined by the presence or absence of cognitive dysfunction in Parkinson’s disease (PD) patients. Patients in the cognitively intact subgroup showed higher levels of serum PACAP than in the cognitive dysfunction subgroup. **(B)** Serum VIP levels in different subgroups defined by the presence or absence of cognitive dysfunction in PD patients. Patients in the cognitively intact subgroup showed higher levels of serum VIP than in the cognitive dysfunction subgroup. **(C)** Serum VIP levels between the anxiety and non-anxiety subgroups in PD patients. Patients in the non-anxiety subgroup showed higher levels of serum VIP than those in the anxiety subgroup. **(D)** Serum VIP levels between the depression and non-depression subgroups in PD patients. Patients in the non-depression subgroup showed higher levels of serum VIP than those in the depression subgroup. Values are presented as the mean ± SD; NS, no significance; ^∗∗^*P* < 0.05.

This further analysis found that lower serum PACAP levels were detected in the cognitive dysfunction subgroup than in the cognitively intact subgroup [(61.87 ± 32.66) pg/ml vs. (84.51 ± 47.59) pg/ml, *P* < 0.05], while serum VIP levels in the cognitive dysfunction subgroup were lower than in the cognitively intact subgroup [(108.04 ± 14.79) pg/ml vs. (110.48 ± 15.85) pg/ml], there was no statistically significant difference (*P* > 0.05). Additionally, we found that lower serum VIP levels were detected in the anxiety subgroup and depression subgroup than in the non-anxiety subgroup and non-depression subgroup [(107.45 ± 15.40) pg/ml vs. (116.41 ± 13.67) pg/ml, *P* < 0.05]; [(104.45 ± 15.26) pg/ml vs. (113.43 ± 14.52) pg/ml, *P* < 0.05]. However, we found no statistically significant difference in VIP levels between the cardiovascular symptom subgroup and the negative cardiovascular symptom subgroup in PD patients. The values are presented as the mean and standard deviation (Figure 6).

## Discussion

In recent years, some progress has been made in studies on the neuroprotective effects of some newly-discovered brain-gut peptides, such as pituitary adenylate cyclase activating polypeptide, vasoactive intestinal polypeptide and et al. PACAP is a neuropeptide isolated from sheep hypothalamus, which has potential to increase adenylate cyclase activity in the pituitary gland (Miyata et al., 1989) and VIP, which has potent and diverse biological action, was isolated from the small intestine of the hog. It is now completely clear that PACAP is packaged and released throughout the peripheral autonomic nervous system. In the sympathetic nervous system, it acts as a primary neurotransmitter and in the parasympathetic it appears to play a neuromodulatory role. PACAP is released at significant levels throughout the periphery upon stressor activation. Likewise, VIP is also released throughout the peripheral nervous system, including in the gut and other organs. The role of PACAP and VIP and in neurodegenerative murine models has been widely explored, particularly in PD models, which exhibits anti-inflammatory and neuroprotective effects. Therefore, we measured the serum levels of PACAP and VIP in PD patients and analyze the relationship with non-motor symptoms for the first time.

To our knowledge, there is only a single report describing PACAP levels in the CSF of PD patients. Han and colleagues included only eight PD patients and found that PACAP levels were not significantly different in the CSF between the PD group and the healthy control group (Han et al., 2014), while no study has been found to measure VIP levels in PD patients. Of interest, our study first found a reduction in peripheral PACAP and VIP levels in PD patients. In addition, after H-Y classification, PACAP and VIP levels were significantly lower in PD patients with early, medium, and advanced stages than in healthy controls, and were gradually reduced with progression of the disease. We consider the following reasons for the observed decrease in serum PACAP and VIP levels in PD patients. First, PACAP is discovered as a hypothalamic peptide (Miyata et al., 1989). Moreover, a recent study found that the precursor of PACAP mRNA in the rat brain is present in different brain areas, especially the highest concentrations of PACAP in the hypothalamic area. As PD progresses, the hypothalamus shows varying degrees of damage (Jellinger, 2002). Collectively, endogenous PACAP levels may be reduced in the serum of PD patients. Second, PACAP and VIP act as anti-inflammatory cytokines and neuroprotective molecules, and the levels may be slightly elevated at the early stage of the disease to protect dopaminergic neurons from degeneration, but when the endogenesis of anti-inflammatory cytokines and neuropeptides such as PACAP or VIP is overwhelmed by an excessive inflammatory response, microglia initiate neuronal death and drive the progressive nature of PD (Wu et al., 2014). Therefore, the above analysis may explain the decrease in peripheral levels. Additionally, (Bukovics et al., 2014) found that plasma and CSF levels of PACAP display parallel patterns in traumatic brain injury survivors. Therefore, it is suggested that PACAP in peripheral blood is derived from the central nervous system (Jiang et al., 2016) and may easily be due to injury-activated release of peripheral PACAP into the serum. The same scenario is also likely for VIP. VIP is released centrally, but has many peripheral sources as well. Of course, further studies are needed to determine whether mRNA expression of PACAP and VIP in the CSF and periphery blood are decreased in PD models and patients.

This study shows that patients with PD have a significant negative correlation between PACAP levels and disease duration, suggesting that decreased serum PACAP levels may play an important role in the progression of this age-related disease, consistent with previous findings (Feher et al., 2018). Additionally, we found a negative correlation between serum VIP levels and UPDRS III scores. Not surprisingly, we found a positive correlation between serum levels of VIP and PACAP regardless of whether they were grouped. Previous studies (Hirabayashi et al., 2018) found that the structures of these two neuropeptides have high homology (∼68% amino acid identity) and exert their action through two common receptors, VPAC1 and VPAC2, while PACAP has a wider range of influence due to an additional specific receptor, PAC1. Thus, the mechanisms of the neuroprotective effects of PACAP and VIP, as well as the signaling pathways, are almost identical (Dong et al., 2019) and our study found that there was a positive correlation between PACAP and VIP levels in the serum. Additionally, serum PACAP and VIP levels showed moderate Se and Sp in differentiating PD patients from controls, which provide a basis for the diagnosis of PD.

Subsequently, we classified PD non-motor symptoms based on the NMSS and analyzed the correlation between non-motor symptoms and PACAP and VIP levels in PD patients. We found that the serum PACAP levels of PD were mainly correlated with scores for item five, assessing Attention/memory. In particular, we found significant differences in the PACAP levels between the cognitive dysfunction subgroup and the cognitively intact subgroup. Therefore, it is speculated that the occurrence of cognitive dysfunction in PD patients has a certain relationship with the reduction of PACAP levels. Clinically, although PD has long been known to be characterized by motor disturbances, cognitive dysfunction is also common in patients and is often manifested as dementia, deficits in learning and attention, or impaired executive function among others (Soles-Tarres et al., 2020). This disorder is often mediated by the extracellular or intracellular accumulation of protein aggregates that disrupt synaptic plasticity, leading to neuronal dysfunction and/or neuronal death. Several mechanisms have been involved in neuronal plasticity disturbance and neuronal cell death, such as inflammation, excitotoxicity, oxidative stress, and neurotrophic deprivation. Although human studies of PACAP are in the preliminary phase, animal studies have shown beneficial effects of PACAP on cognition and memory. Some data in animal models show that the lack of PACAP is associated with cognitive decline. Indeed, PACAP deficient mice display impaired recognition memory (Shibasaki et al., 2015). Moreover, animal studies have shown that an exogenous increase in PACAP may rescue 6-OHDA-induced degenerating dopamine neurons (Reglodi et al., 2004). Deguil et al. (2010) found that pretreatment with PACAP by intravenous injections can partially protect against the loss of TH-positive neurons and improve learning and memory in MPTP-injected mice. Based on these findings, we could infer that the decrease in PACAP levels may be one of the causes of related learning and memory deficits.

We also found that the serum VIP levels of PD and some non-motor symptoms, such as cardiovascular symptoms and mood/cognition disorders, had a certain correlation, the most important being mood/cognition disorders. The remaining non-motor symptoms have not yet been found to be relevant or different. Further analysis found that serum VIP levels were not significantly different between the negative cardiovascular symptom subgroup and the cardiovascular symptom subgroup, whereas lower serum VIP levels were detected in the anxiety subgroup and depression subgroup than in the non-anxiety subgroup and non-depression subgroup. Therefore, it is speculated that the occurrence of mood disorders in PD patients has a certain relationship with internal VIP levels. Mood disorders are now considered among the main non-motor symptoms of PD and have a serious impact on quality of life (Rieu et al., 2016). Among these symptoms, the prevalence of depression ranges from 20 to 40%, and the prevalence of anxiety varies from 8 to 25% (Aarsland et al., 2009; Gallagher and Schrag, 2012). Some studies have found that symptoms of depression and anxiety are more common in patients with PD than in the general population and are related to signs of inflammation (Lindqvist et al., 2012). Decreased VIP levels have been found in patients with major depression, which has also been found in PD patients. Moreover, the lower VIP levels are consistent with reduced parasympathetic tone that has been reported in depression (Gjerris et al., 1984), considering VIP levels probably involved in the pathobiology of affective disorders. A study by Lindqvist et al. (2012) found that depression scores and anxiety scores are significantly associated with pro-inflammatory cytokines. Because of the anti-inflammatory effects of VIP, we speculate that the high VIP content in PD patients who are not accompanied by depression and anxiety symptoms may reduce the release of pro-inflammatory mediators by activated microglia through these effects, thereby reducing the occurrence of mood disorders. In addition, it was found in a 6-OHDA-induced PD rat model that intraperitoneal injection of VIP can increase the number of 5-hydroxytryptamine (5-HT)-positive cells and the relative expression of mRNA in the dorsal raphe nucleus (DRN) and reduce the expression of depression-related neuroendocrine hormones (Wang et al., 2015). A large number of studies have confirmed that the decreased release of the monoamine neurotransmitter serotonin 5-HT in the brain may be one of the causes of clinical depressive symptoms (Gavioli and Calo, 2013), and DRN is the nucleus with the largest distribution of 5-HT neurons and is closely related to stress-related mental disorders such as anxiety and depression (Vasudeva et al., 2011). Therefore, we infer that VIP may have a potential therapeutic effect on patients with PD associated with mood disorders, which also provides a new research direction for the treatment of neurodegenerative diseases with mood disorders. In addition, VIP levels are reduced in PD, and these patients may have an increased risk of cardiovascular disease. Therefore, we can infer that VIP can reduce the incidence of cardiovascular adverse events and reduce cardiovascular symptoms to a certain extent by inhibiting the release of pro-inflammatory factors.

Our study had several limitations. First, patients were collected from a single clinical center. Hence, multicentre studies with more patients will be required to further investigate the changes in serum PACAP and VIP levels. Second, whether peripheral PACAP and VIP levels reflect central-related material activity remains controversial. To further clarify the clinical significance of PACAP and VIP levels in serum, it is necessary to investigate whether PACAP and VIP levels in serum and in CSF move in parallel. Third, in contrast to cross-sectional studies, a more accurate and appropriate approach would be to assess PACAP and VIP levels in follow-up studies.

## Conclusion

Our study found a reduction in the levels of PACAP and VIP in PD for the first time. At the same time, PACAP levels in relation to cognitive function and VIP levels in relation to mood disorders were proven. These findings suggest potential underlying link between serum PACAP and VIP levels and the non-motor symptoms in PD patients. Therefore, PACAP and VIP levels can be used to evaluate PD-related non-motor symptom–cognitive function and mood disorders, respectively.

## Data Availability Statement

The original contributions presented in the study are included in the article/supplementary material, further inquiries can be directed to the corresponding author.

## Ethics Statement

The studies involving human participants were reviewed and approved by the Ethics Committee of the Henan Provincial People’s Hospital. The patients/participants provided their written informed consent to participate in this study.

## Author Contributions

SYH and JM completed the topic selection and study design. SYH conducted the experiments. SH, DL, ZZ, and JZ conducted the literature search, acquisition of data, and study supervision. ML, ZW, WS, and XS performed the statistical analysis and interpretation of data. SYH and JM drafted the manuscript. All authors contributed to the manuscript and gave final approval.

## Conflict of Interest

The authors declare that the research was conducted in the absence of any commercial or financial relationships that could be construed as a potential conflict of interest.

## Publisher’s Note

All claims expressed in this article are solely those of the authors and do not necessarily represent those of their affiliated organizations, or those of the publisher, the editors and the reviewers. Any product that may be evaluated in this article, or claim that may be made by its manufacturer, is not guaranteed or endorsed by the publisher.
